# Ethnic disparities in estimated cardiovascular disease risk in Amsterdam, the Netherlands: The HELIUS study

**DOI:** 10.1007/s12471-018-1107-3

**Published:** 2018-04-11

**Authors:** W. Perini, M. B. Snijder, R. J. G. Peters, A. E. Kunst

**Affiliations:** 1 Department of Public Health, Academic Medical Center Amsterdam, Amsterdam, The Netherlands; 2 Department of Cardiology, Academic Medical Center Amsterdam, Amsterdam, The Netherlands; 3 Department of Clinical Epidemiology, Biostatistics and Bioinformatics, Academic Medical Center Amsterdam, Amsterdam, The Netherlands

**Keywords:** Cardiovascular disease, Ethnic groups, Primary prevention, Hypertension, Dyslipidaemia, HELIUS study

## Abstract

**Background:**

Ethnic differences have been reported in cardiovascular disease (CVD) risk factors. It is still unclear which ethnic groups are most at risk for CVD when all traditional CVD risk factors are considered together as overall risk.

**Objectives:**

To examine ethnic differences in overall estimated CVD risk and the risk factors that contribute to these differences.

**Design:**

Using data of the multi-ethnic HELIUS study (HEalthy LIfe in an Urban Setting) from Amsterdam, we examined whether estimated CVD risk and risk factors among those eligible for CVD risk estimation differed between participants of Dutch, South Asian Surinamese, African Surinamese, Ghanaian, Turkish and Moroccan origin. Using the Systematic COronary Risk Evaluation (SCORE) algorithm, we estimated risk of fatal CVD and risk of fatal plus non-fatal CVD. These risks were compared between ethnic groups via age-adjusted linear regression analyses.

**Results:**

The SCORE algorithm was applicable to 9,128 participants. Relative to the fatal CVD risk of participants of Dutch origin, South Asian Surinamese participants showed a higher fatal CVD risk, Ghanaian males a lower fatal CVD risk, and participants of other ethnic origins a similar fatal CVD risk. For fatal plus non-fatal CVD risk, African Surinamese and Turkish men also showed a higher risk. When diabetes was incorporated in the CVD risk algorithm, all but Ghanaian men showed a higher CVD risk relative to the participants of Dutch origin (betas ranging from 0.98–3.10%). The CVD risk factors that contribute the most to these ethnic differences varied between ethnic groups.

**Conclusion:**

Ethnic minority groups are at a greater estimated risk of fatal plus non-fatal CVD relative to the group of native Dutch. Further research is necessary to determine whether this will translate to ethnic differences in CVD incidence and, if so, whether ethnic-specific CVD prevention strategies are warranted.

**Electronic supplementary material:**

The online version of this article (10.1007/s12471-018-1107-3) contains supplementary material, which is available to authorized users.

## What’s new

- Estimated fatal cardiovascular disease risk is similar between the population of Dutch origin and most ethnic minority groups
- However, for non-fatal cardiovascular disease the estimated risk is higher among most ethnic minority groups relative to the group of Dutch origin
- Ethnic differences in cardiovascular disease risk are especially large when diabetes is incorporated in cardiovascular risk estimation

## Introduction

Cardiovascular disease (CVD) is one of the leading causes of mortality and morbidity and is distributed unequally among ethnic groups [1–4]. Among several ethnic minority groups, studies have reported a high burden of CVD [1–4]. Therefore, there has been a call for multi-ethnic research on CVD prevention [1].

Targeted CVD prevention strategies are based on identifying high-risk individuals [1, 5]. CVD risk is often defined as the ten-year risk of mortality due to coronary heart disease or stroke, which can be estimated by CVD risk algorithms based on the occurrence of CVD risk factors [1, 5]. Based on the estimated CVD risk, the initiation of preventive intervention can be tailored to the individual [1, 5].

Ethnic groups may differ in the prevalence of individual CVD risk factors and, consequently, in estimated CVD risk [1, 6, 7]. For example, South Asians are predisposed to developing dyslipidaemia and diabetes, whereas Ghanaians have a lower prevalence of smoking, but a higher prevalence of hypertension compared with most other ethnic groups [8–10]. In addition, the prevalence of individual CVD risk factors is generally expected to be relatively high among ethnic minority groups due to a high exposure to conditions associated with CVD risk (e. g. low socioeconomic status and ethnic discrimination) [11–13].

Although previous studies have examined ethnic disparities in isolated CVD risk factors, European studies regarding ethnic disparities in overall estimated CVD risk are lacking. Therefore, it is unknown which ethnic groups may be most at risk for CVD when all CVD risk factors are considered together, which is why we do not know among which ethnic groups preventive intervention for CVD may be the most warranted. In this study, we aim to estimate CVD risk based on the presence of CVD risk factors via the Systematic COronary Risk Evaluation [SCORE] algorithm and determine whether ethnic disparities in estimated CVD risk occur. To better understand these ethnic disparities, we will examine ethnic differences in the individual CVD risk factors used to estimate CVD risk.

## Methods

During the HELIUS (Healthy Life in an Urban Setting) study, a large-scale, multi-ethnic cohort study on health and health care utilisation among different ethnic groups living in Amsterdam, the Netherlands, data were obtained via questionnaires, physical examinations and biological samples. The aims and design of the HELIUS study have been published [14]. In brief, participants between 18–70 years of age living in Amsterdam were randomly sampled via the municipality register, after stratification by ethnicity. Both questionnaire and physical examination data were obtained among 22,165 participants. The study protocols were approved by the AMC Ethical Review Board, and all participants provided written informed consent.

### Ethnicity

Ethnicity was defined according to the country of birth of the participant as well as that of his/her parents [15]. A participant was considered as of non-Dutch ethnic origin if he/she was born abroad and had at least one parent born abroad (first generation); or he/she was born in the Netherlands but both his/her parents were born abroad (second generation) [15]. Surinamese subgroups were classified according to self-reported ethnic origin. Approximately 80% are either of Creole (African) origin or of Hindustani (South Asian) origin.

### Cardiovascular risk

CVD risk was estimated via the SCORE algorithm based on total cholesterol/high-density lipoprotein cholesterol ratio (TC/HDL) for low-risk countries [16]. This algorithm estimates the ten-year risk of mortality due to coronary heart disease or stroke based on gender, age, blood pressure, TC/HDL and smoking status (yes/no) among those who are 40–65 years of age without diabetes or prior CVD [16]. In addition, we estimated the total cardiovascular risk (mortality plus morbidity) using the Dutch SCORE algorithm (dSCORE), which is derived from the same algorithm, but includes an age-dependent conversion factor between 3 and 5, with a higher conversion factor among younger people, to estimate the ten-year risk of fatal plus non-fatal CVD [5]. The dSCORE algorithm also adds fifteen years to the calendar age among those with diabetes and includes a specific algorithm to estimate CVD risk for participants above the age of 65. In addition, we also estimated total CVD risk using the age-specific conversion factors described by Jorstad et al. [17], which are based on the European Prospective Investigation into Cancer and Nutrition cohort (eSCORE).

Smoking status and prior CVD were assessed by questionnaire. Blood pressure was measured in duplicate using a validated automated digital blood pressure device (WatchBP Home; Microlife AG) on the left arm in a seated position after the person had been seated for at least 5 min. Fasting blood samples were drawn, and glucose and TC/HDL were determined. Participants were considered to have diabetes if they reported a diabetes diagnosis, use of glucose-lowering medication, or in case of a fasting glucose equal or above 7.0 mmol/l [18].

### Study population

We excluded participants with a Javanese Surinamese (*n* = 233), ‘other Surinamese’ (*n* = 267) or unknown/another ethnicity (*n* = 48). Furthermore, we excluded participants based on missing data regarding cardiovascular risk factors (i. e. age, sex, smoking status, blood pressure, lipid profile or diabetes status) (*n* = 372) and due to not being eligible for CVD risk estimation using the SCORE algorithm based on age below 40 (*n* = 7581), age above 65 (*n* = 868), the presence of diabetes (*n* = 1,911) or prior myocardial infarction and/or stroke (*n* = 1,756). We also excluded one participant with unlikely high cholesterol values. This resulted in a study population of 9,128 participants.

### Statistical analyses

Analyses were stratified by gender. We used multiple linear regression analyses to determine ethnic differences in estimated CVD risk, adjusted for age. Because of non-linear associations between age and estimated CVD risk, age was stratified into five-year age categories. Next, we determined ethnic differences in CVD risk factors using logistic regression for dichotomous, and linear regression for continuous CVD risk factors. Similar analyses were performed using the dSCORE and the eSCORE as outcome variable in the same study sample.

For sensitivity analyses, we excluded participants using antihypertensive and/or lipid-lowering medication (*n* = 1,639) and repeated all analyses. Furthermore, we repeated the dSCORE analyses including participants who are not eligible for CVD risk estimation using the SCORE algorithm, but who are eligible for CVD risk estimation using the dSCORE (in accordance to the current clinical practice in Dutch primary care). We therefore included participants aged 25–55 with diabetes (*n* = 863) and participants without diabetes above the age of 65 (*n* = 499).

## Results

Ethnic groups differed in age, occurrence of CVD risk factors, and use of preventive medication (Tab. 1). Ghanaian participants and Moroccan women showed a relatively low prevalence of smoking. African Surinamese and Ghanaian participants showed a high mean systolic blood pressure, but favourable lipid profiles.

**Table 1:** General characteristics by ethnicity

	Dutch	South-Asian Surinamese	African Surinamese	Ghanaian	Turkish	Moroccan
*Men*						
*N*	997	449	811	484	567	519
Age (years)	52.5 (7.4)	50.1 (6.4)	52.6 (6.6)	51.0 (6.0)	48.7 (5.6)	49.6 (6.5)
Smoking (%)	21.9	36.1	45.5	7.9	39.5	25.6
SBP (mm Hg)	130.6 (16.1)	132.4 (16.6)	135.2 (17.3)	140.8 (17.5)	127.8 (14.8)	128.2 (14.3)
Antihypertensive treatment (%)	8.6	13.4	16.2	26.4	7.2	4.6
TC (mmol/l)	5.42 (9.96)	5.35 (0.93)	5.04 (0.98)	5.17 (0.99)	5.20 (0.91)	4.95 (0.84)
HDL (mmol/l)	1.40 (0.39)	1.18 (0.29)	1.39 (0.38)	1.55 (0.42)	1.14 (0.29)	1.17 (0.28)
TC/HDL	4.13 (1.25)	4.75 (1.25)	3.85 (1.19)	3.54 (1.05)	4.82 (1.41)	4.44 (1.17)
Lipid-lowering treatment (%)	5.6	12.5	3.9	6.2	6.3	2.5
*Women*						
*N*	1,259	690	1,264	700	651	737
Age (years)	52.6 (7.2)	50.9 (6.4)	51.9 (6.6)	48.6 (5.6)	48.0 (5.8)	49.0 (6.4)
Smoking (%)	21.3	17.7	22.5	2.4	25.5	2.3
SBP (mm Hg)	122.2 (16.4)	123.4 (20.0)	132.2 (18.0)	137.8 (18.8)	124.4 (16.7)	123.0 (15.83)
Antihypertensive treatment (%)	10.2	17.4	27.5	31.4	14.6	7.5
TC (mmol/l)	5.50 (1.02)	5.33 (0.93)	5.15 (0.94)	5.13 (0.95)	5.21 (0.93)	5.02 (0.86)
HDL (mmol/l)	1.78 (0.44)	1.50 (0.39)	1.66 (0.45)	1.75 (0.45)	1.44 (0.35)	1.45 (0.34)
TC/HDL	3.28 (1.02)	3.75 (1.04)	3.30 (1.00)	3.09 (0.85)	3.83 (1.10)	3.61 (0.95)
Lipid-lowering treatment (%)	4.0	9.9	4.5	3.9	4.0	2.0

Data are presented as mean (standard deviation) or percentages

*SBP* systolic blood pressure, *HDL* high-density lipoprotein, *TC* total cholesterol

Fatal CVD risk as estimated by SCORE was similar among most ethnic groups (Tab. 2). However, relative to the native Dutch, South Asian Surinamese participants showed higher estimated fatal CVD risk, while Ghanaian men showed lower estimated fatal CVD risk. When using the dSCORE algorithm, estimated fatal plus non-fatal CVD risk was also higher among African Surinamese participants and Turkish men. When using the eSCORE algorithm, estimated CVD risk was higher relative to the native Dutch group among all ethnic groups except for Ghanaian men and Moroccan participants who showed similar estimated CVD risk compared with the participants of Dutch origin.

**Table 2:** Age-adjusted mean estimated CVD risk and age-adjusted ethnic differences (ref. Dutch) in CVD risk as estimated by SCORE, dSCORE and eSCORE

	SCORE^a^		dSCORE^b^		eSCORE^c^	
	Mean	Difference	Mean	Difference	Mean	Difference
*Men*						
Dutch	1.59	0	6.18	0	15.63	0
South-Asian Surinamese	1.95	* 0.30 (0.17; 0.44)*	7.70	* 1.39 (0.89; 1.89)*	19.63	* 3.73 (2.58; 4.87)*
African Surinamese	1.66	0.06 (−0.05; 0.17)	6.67	* 0.47 (0.06; 0.88)*	17.04	* 1.38 (0.43; 2.32)*
Ghanaian	1.40	*−0.21 (−0.34; −0.08)*	5.69	*−0.53 (−1.01; −0.04)*	14.62	−1.06 (−2.17; 0.05)
Turkish	1.68	0.12 (−0.01; 0.24)	6.91	* 0.77 (0.31; 1.24)*	17.89	* 2.18 (1.11; 3.25)*
Moroccan	1.58	−0.02 (−0.15; 0.11)	6.28	0.08 (−0.39; 0.56)	15.93	0.25 (−0.84; 1.35)
*Women*						
Dutch	0.47	0	1.73	0	7.25	0
South-Asian Surinamese	0.54	* 0.06 (0.02; 0.10)*	2.08	* 0.32 (0.18; 0.45)*	8.90	* 1.62 (1.12; 2.12)*
African Surinamese	0.51	0.03 (−0.00; 0.06)	1.89	* 0.20 (0.08; 0.31)*	8.45	* 1.25 (0.83; 1.68)*
Ghanaian	0.45	−0.01 (−0.05; 0.04)	1.72	0.07 (−0.06; 0.21)	8.16	* 0.92 (0.41; 1.43)*
Turkish	0.50	0.03 (−0.02; 0.07)	1.83	0.16 (0.02; 0.30)	8.46	* 1.11 (0.59; 1.63)*
Moroccan	0.44	−0.01 (−0.05; 0.03)	1.75	0.03 (−0.10; 0.17)	7.38	0.16 (−0.34; 0.66)

Italic indicates statistical significant difference from the Dutch

*CVD* cardiovascular disease, *SCORE* systematic coronary risk evaluation, *dSCORE* Dutch SCORE, *eSCORE* European Prospective Investigation into Cancer and Nutrition cohort SCORE

^a^Ten-year risk (%) of fatal CVD as estimated by SCORE

^b^Ten-year risk (%) of fatal plus nonfatal CVD as estimated by dSCORE

^c^Ten-year risk (%) of fatal plus nonfatal CVD as estimated by eSCORE

Clusters of risk factors differed between the ethnic groups (Supplementary Table 1). Relative to the native Dutch, Turkish and Moroccan men showed a dyslipidaemic CVD risk profile (i. e. higher prevalence of dyslipidaemia but lower prevalence of hypertension), Ghanaian and African Surinamese participants showed a hypertensive CVD risk profile (i. e. higher prevalence of hypertension but lower prevalence of dyslipidaemia) and South Asian Surinamese participants and Moroccan and Turkish women showed a combined risk profile of both hypertension and dyslipidaemia. Among men, all ethnic minority groups showed a higher prevalence of smoking except for Ghanaian men whereas ethnic minority women showed a similar or lower prevalence of smoking relative to the native Dutch group. Results were similar when we excluded participants taking antihypertensive medication and/or lipid-lowering medication (Supplementary Tables 2 and 3).

When the dSCORE was used among all participants eligible for CVD risk estimation according to Dutch clinical guidelines (i. e. including participants with diabetes aged 25–55 and participants without diabetes above the age of 65), estimated fatal plus non-fatal CVD risk was higher among all ethnic groups relative to the native Dutch group except among Ghanaian men, who showed a similar estimated CVD risk (Tab. 3). Ethnic differences in CVD risk factors were similar to those found among participants only eligible for CVD risk estimation using SCORE (Supplementary Table 1).

**Table 3:** Among all participants eligible for CVD risk estimation using the dSCORE: age-adjusted mean and beta (CI) for ethnic differences in CVD risk^a^ plus beta (CI) or odds ratio (CI) for ethnic differences in CVD risk factors

	Dutch (ref)	South-Asian Surinamese	African Surinamese	Ghanaian	Turkish	Moroccan
*Men*						
Mean dSCORE	8.01	11.28	9.28	8.33	9.95	9.35
dSCORE difference	0	* 3.10 (2.30; 3.91)*	* 1.14 (0.46; 1.82)*	0.53 (−0.26; 1.33)	* 1.93 (1.16; 2.70)*	* 1.13 (0.35; 1.91)*
Smoking	1	* 2.08 (1.66; 2.61)*	* 2.85 (2.36; 3.46)*	* 0.28 (0.20; 0.39)*	* 2.16 (1.74; 2.68)*	1.10 (0.87; 1.39)
SBP (mm Hg)	0	* 3.04 (1.39; 4.69)*	* 4.88 (3.49; 6.27)*	*11.05 (9.43; 12.67)*	−0.33 (−1.90; 1.25)	−0.66 (−2.25; 0.93)
TC/HDL	0	* 0.52 (0.39; 6.53)*	*−0.26 (−0.37; −0.15)*	*−0.60 (−0.73; −0.47)*	* 0.62 (0.50; 0.75)*	* 0.28 (0.15; 0.41)*
Diabetes	1	* 6.90 (4.35; 10.95)*	* 3.10 (1.92; 4.99)*	* 6.36 (4.00; 10.12)*	* 4.90 (3.08; 7.78)*	* 4.78 (2.99; 7.65)*
*Women*						
Mean dSCORE	3.25	6.18	4.37	3.87	5.28	5.73
dSCORE difference	0	* 2.64 (1.82; 3.45)*	* 0.98 (0.28; 1.68)*	* 1.10 (0.25; 1.95)*	* 1.83 (0.97; 2.69)*	* 2.33 (1.51; 3.14)*
Smoking	1	* 0.75 (0.60; 0.94)*	1.03 (0.86; 1.24)	* 0.09 (0.06; 0.14)*	1.08 (0.87; 1.34)	* 0.07 (0.04; 1.11)*
SBP (mm Hg)	0	* 9.40 (7.83; 10.76)*	*10.59 (9.34; 11.84)*	*18.62 (17.10; 20.14)*	* 6.14 (4.59; 7.77)*	* 4.14 (2.69; 5.60)*
TC/HDL	0	* 0.51 (0.42; 0.60)*	0.06 (−0.02; 0.13)	*−0.12 (−0.22; −0.03)*	* 0.63 (0.53; 0.72)*	* 0.41 (0.32; 0.49)*
Diabetes	1	*20.03 (9.66; 41.51)*	*11.56 (5.59; 23.92)*	*14.60 (6.98; 30.57)*	*16.42 (7.86; 34.30)*	*21.87 (10.60; 45.13)*

Italic indicates statistical significant difference from the Dutch

*CI* confidence interval, *CVD* cardiovascular disease, *dSCORE* Dutch systematic coronary risk evaluation,* SBP* systolic blood pressure, *TC/HDL* total cholesterol/high-density cholesterol

^a^Ten-year risk (%) of fatal plus nonfatal CVD as estimated by dSCORE

## Discussion

### Key findings

Fatal CVD risk as estimated by SCORE is higher among South Asian Surinamese men and women, lower among Ghanaian men and similar among all other ethnic groups relative to the native Dutch group. When fatal plus non-fatal CVD risk is estimated using traditional SCORE CVD risk factors plus diabetes, all ethnic minority groups show higher estimated CVD risk relative to the native Dutch group, except for Ghanaian men who show a similar CVD risk. Ethnic groups differ in whether they show a dyslipidaemic, hypertensive or combined CVD risk profile relative to the participants of Dutch origin.

### Strengths and limitations

Due to the large multi-ethnic sample size, we were able to determine ethnic differences in estimated CVD risk, stratified for gender. Furthermore, this study compared ethnic differences in both fatal CVD risk and fatal plus non-fatal CVD risk.

A potential weakness is the application of the SCORE algorithm to different ethnic groups, as SCORE is validated mainly among ethnic majority populations [1, 19]. The SCORE algorithm may underestimate or overestimate CVD risk among ethnic minority groups [1]. Moreover, recent studies have identified CVD risk factors which may substantially increase CVD risk independently of traditional CVD risk factors (e. g. psoriasis, human immunodeficiency virus infection or lipoprotein (a)) [1, 20, 21]. These factors may not be distributed equally among ethnic groups [22, 23]. Thus, ethnic differences in true CVD risk may differ from the risk differences as estimated in our study.

As this study was conducted in Amsterdam, results may not be generalisable to ethnic minority populations residing in other cities or countries. Studies in other settings are needed to identify possible common patterns. For example, a study from Norway described a similar higher estimated CVD mortality risk among foreign-born participants relative to participants born in Norway [24].

### Discussion of key findings

In contrast to our results, an earlier nation-wide study showed lower CVD mortality among the Moroccan population relative to the population of Dutch origin [2, 3]. This discrepancy may be attributable to risk factors not included in SCORE, but may also reflect that, in this nation-wide study, the ethnic differences in CVD mortality were observed within older participants and were mainly driven by the lifetime exposures to risk factors of these participants.

Among Ghanaians, estimated CVD risk was relatively low compared with other ethnic groups despite a higher prevalence of systolic hypertension. This may be due to a low prevalence of smoking. Earlier nationwide analysis also showed a lower incidence of acute myocardial infarction among Ghanaians compared with the native Dutch [3]. However, estimated CVD risk among Ghanaians may underestimate true lifetime CVD risk, for example due to an early onset of hypertension and thus a longer risk exposure, which the SCORE algorithm does not take into account [25]. If so, current guidelines for antihypertensive treatment may not be optimal for the Ghanaian population.

Our final analyses included diabetes as a CVD risk factor by adding 15 years to the calendar age among those with diabetes, which may result in a great increase in estimated CVD risk [5]. Considering the relatively high prevalence of diabetes among ethnic minority groups, it is not surprising that considering diabetes resulted in greater ethnic disparities in estimated CVD risk. These disparities may be even greater when considering the relatively young onset of type 2 diabetes among ethnic minority groups and thus the relatively long exposure to hyperglycaemia among these groups [10].

This study found greater ethnic differences in CVD risk when CVD risk is estimated based on fatal plus non-fatal CVD instead of fatal CVD alone. This may suggest that ethnic disparities in CVD risk already occur at a young age, as conversion factors to estimate non-fatal CVD risk based on fatal CVD risk are higher at a young age. Accordingly, previous studies reported a younger onset of CVD risk and a higher prevalence or mean of CVD risk factors at a young age among ethnic minority groups relative to the host group [10, 26, 27].

Earlier studies from several countries have reported relatively low access to health care facilities among ethnic minority groups [28]. Therefore, these groups may benefit less from efforts to reduce CVD risk. However, as a result from the universally accessible health care system, ethnic differences in health care access may not be as pervasive in the Netherlands [29, 30]. Thus, in the Netherlands, we do not expect that access to health care facilities will contribute to ethnic disparities in the potential effect of CVD prevention strategies on CVD risk.

## Conclusion

Although estimated fatal CVD risk that is based on traditional SCORE risk factors is generally similar between ethnic groups, estimated fatal plus non-fatal CVD risk that is based on traditional SCORE risk factors plus diabetes is generally higher among ethnic minority groups relative to the native Dutch. The CVD risk factors contributing most to the increased estimated CVD risk differ between ethnic groups. Further research is necessary to determine whether these ethnic differences in estimated CVD risk will translate to ethnic differences in CVD incidence and, if so, whether ethnic-specific CVD prevention strategies are warranted.

## Caption Electronic Supplementary Material

Supplementary Tables 1–3
