# Supplementary material for: Ethnic disparities in estimated cardiovascular disease risk in Amsterdam, the Netherlands: The HELIUS study
Source: Neth Heart J. 2018 Apr 11;26(5):252–62. doi: 10.1007/s12471-018-1107-3 (PMC5910313; doi:10.1007/s12471-018-1107-3)
Supplement: Supplementary file 1 — Supplementary Tables 1–3 [file 12471_2018_1107_MOESM1_ESM.doc]

**Supplementary Table 1 Odds ratio (95% CI) or beta (95% CI) in CVD risk factors between the Dutch group and ethnic minority groups, adjusted for age**

|  | Dutch  (ref) | South-Asian  Surinamese | African  Surinamese | Ghanaian | Turkish | Moroccan |
| --- | --- | --- | --- | --- | --- | --- |
| Men |  |  |  |  |  |  |
| Smoking | 1 | **1.98  (1.55; 2.54)** | **2.99  (2.44; 3.66)** | **0.30  (0.21; 0.43)** | **2.27  (1.81; 2.86)** | 1.21  (0.94; 1.55) |
| SBP | 0 | **3.01  (1.33; 4.87)** | **4.53  (3.06; 6.00)** | **10.97  (9.25; 12.69)** | -0.83  (-2.48; 0.83) | -0.91  (-2.60; 0.79) |
| TC/HDL | 0 | **0.60  (0.46; 0.74)** | -0.28  (-0.40; -0.17) | -0.60  (-0.74; -0.47) | **0.67  (0.53; 0.78)** | **0.28  (0.15; 0.41)** |
| Women |  |  |  |  |  |  |
| Smoking | 1 | **0.76  (0.60; 0.96)** | 1.06  (0.88; 1.28) | **0.08  (0.05; 0.14)** | 1.11  (0.89; 1.40) | **0.08  (0.05; 0.13)** |
| SBP | 0 | **8.45  (6.91; 10.04)** | **10.49  (9.18; 11.80)** | **18.71  (17.13; 20.28)** | **5.68  (4.06; 7.30)** | **3.57  (2.02; 5.12)** |
| TC/HDL | 0 | **0.49  (0.40; 0.59)** | 0.04  (-0.04; 0.11) | -0.13  (-0.22; -0.04) | **0.62  (0.53; 0.72)** | **0.40  (0.30; 0.48)** |

bold indicates statistical significant difference from the Dutch

*CI* confidence interval, *SPB* systolic blood pressure, *TC/HDL* total cholesterol/high-density cholesterol

**Supplementary Table 2** Age-adjusted mean CVD risk and age-adjusted differences (beta, 95% CI) ethnic differences (ref. Dutch) in CVD risk as estimated by SCORE^1^, dSCORE^2^ and eSCORE^3^ excluding participants using antihypertensive or lipid-lowering medication

|  | SCORE^1^ | | dSCORE^2^ | | eSCORE^3^ | |
| --- | --- | --- | --- | --- | --- | --- |
|  | Mean | Difference | Mean | Difference | Mean | Difference |
| Men |  |  |  |  |  |  |
| Dutch | 1.44 | 0 | 5.69 | 0 | 14.35 | 0 |
| South-Asian  Surinamese | 1.81 | 0.31 (0.18-0.46) | 7.22 | **1.37 (0.84-1.90)** | 18.45 | **3.68 (2.46-4.89)** |
| African  Surinamese | 1.55 | **0.09 (-0.03-2.0)** | 6.19 | **0.53 (0.10-0.96)** | 15.88 | **1.52 (0.54-2.50)** |
| Ghanaian | 1.31 | -0.14 (-0.28-0.00) | 5.35 | **-0.33 (-0.86-0.20)** | 13.79 | -0.63 (-1.84-0.59) |
| Turkish | 1.55 | **0.14 (0.01-0.27)** | 6.34 | **0.78 (0.31-1.25)** | 16.66 | **2.28 (1.20-3.37)** |
| Moroccan | 1.47 | 0.03 (-0.10-0.15) | 5.91 | 0.199 (-0.28-0.67) | 15.07 | 0.61 (-0.58-1.70) |
| Women |  |  |  |  |  |  |
| Dutch | 0.36 | 0 | 1.46 | 0 | 6.44 | 0 |
| South-Asian  Surinamese | 0.44 | 0.02 (-0.02-0.07) | 1.69 | **0.18 (0.05-0.31)** | 7.59 | **1.13 (0.62-1.63)** |
| African  Surinamese | 0.43 | **0.02 (-0.01-0.06)** | 1.66 | **0.16 (0.05-0.28)** | 7.62 | **1.26 (0.83-1.70)** |
| Ghanaian | 0.37 | 0.00 (-0.04-0.04) | 1.52 | 0.09 (-0.06-0.23) | 7.43 | **0.94 (0.41-1.47)** |
| Turkish | 0.37 | 0.00 (-0.04-0.04) | 1.51 | 0.08 (-0.06-0.22) | 7.27 | **0.86 (0.35-1.37)** |
| Moroccan | 0.37 | -0.02 (-0.05-0.02) | 1.53 | 0.00 -0.12-0.13) | 6.53 | 0.11 (-0.36-0.59) |

1: ten-year risk (%) of fatal CVD as estimated by SCORE

2: ten-year risk (%) of fatal plus nonfatal CVD as estimated by dSCORE

3: ten-year risk (%) of fatal plus nonfatal CVD as estimated by eSCORE

bold indicates statistical significant difference from the Dutch

*CVD* cardiovascular disease, *SCORE* systematic coronary risk evaluation, *dSCORE* Dutch SCORE, *eSCORE* European Prospective Investigation into Cancer and Nutrition cohort SCORE

**Supplementary Table 3** Odds ratio (95% CI) or beta (95% CI) in CVD risk factors between the Dutch ethnic group and ethnic minority groups, adjusted for age, excluding participants using antihypertensive or lipid-lowering medication

|  | Dutch  (ref) | South-Asian  Surinamese | African  Surinamese | Ghanaian | Turkish | Moroccan |
| --- | --- | --- | --- | --- | --- | --- |
| Men |  |  |  |  |  |  |
| Smoking | 1 | **2.31  (1.76; 3.03)** | **3.36  (2.69; 4.19)** | **0.34  (0.23; 0.52)** | **2.43  (1.90; 3.10)** | 1.24  (0.95; 1.61) |
| SBP^1^ | 0 | 1.78  (-0.17; 3.72) | **3.97  (2.40; 5.54)** | **9.93  (7.99; 11.87)** | -0.93  (-2.66; 0.81) | -0.76  (-2.50; 0.98) |
| TC/HDL^2^ | 0 | **0.69  (0.54; 0.84)** | -0.25  (-0.37; -0.12) | -0.56  (-0.71; -0.41) | **0.71  (0.57; 0.84)** | **0.35  (0.21; 0.49)** |
| Women |  |  |  |  |  |  |
| Smoking | 1 | 0.80  (0.62; 1.04) | 1.12  (0.91; 1.39) | **0.08  (0.04; 0.15)** | 1.17  (0.92; 1.50) | **0.09  (0.05; 0.15)** |
| SBP^1^ | 0 | **6.42  (4.77; 8.08)** | **9.83  (8.41; 11.24)** | **18.28  (16.53; 20.03)** | **4.72  (3.04; 6.40)** | **3.40  (1.84; 4.95)** |
| TC/HDL^2^ | 0 | **0.59  (0.49; 0.69)** | **0.11  (0.02; 0.20)** | -0.05  (-0.16; 0.79) | **0.69  (0.58; 0.79)** | **0.45  (0.35; 0.54)** |

bold indicates statistical significant difference from the Dutch

*CI* confidence interval, *SPB* systolic blood pressure, *TC/HDL* total cholesterol/high-density cholesterol
